# Preconception Prebiotic and Sitagliptin Treatment in Obese Rats Affects Pregnancy Outcomes and Offspring Microbiota, Adiposity, and Glycemia

**DOI:** 10.3389/fendo.2017.00301

**Published:** 2017-10-30

**Authors:** Carol A. Dennison, Amanda J. Eslinger, Raylene A. Reimer

**Affiliations:** ^1^Faculty of Kinesiology, University of Calgary, Calgary, AB, Canada; ^2^Department of Biochemistry and Molecular Biology, Cumming School of Medicine, University of Calgary, Calgary, AB, Canada

**Keywords:** oligofructose, dipeptidyl peptidase-4 inhibitor, maternal obesity, prepregnancy, gut microbiota, maternal programming

## Abstract

Maternal obesity is associated with increased risk of pregnancy complications and greater risk of obesity in offspring, but studies designed to examine preconception weight loss are limited. The objective of this study was to determine if a combined dietary [oligofructose (OFS)] and pharmacological (sitagliptin) preconception intervention could mitigate poor pregnancy outcomes associated with maternal obesity and improve offspring metabolic health and gut microbiota composition. Diet-induced obese female Sprague-Dawley rats were randomized to one of four intervention groups for 8 weeks: (1) Obese-Control (consumed control diet during intervention); (2) Obese-OFS (10% OFS diet); (3) Obese-S (sitagliptin drug); (4) Obese-OFS + S (combination treatment). Two reference groups were also included: (5) Obese-HFS (untreated obese consumed high fat/sucrose diet throughout study); (6) Lean-Control (lean reference group that were never obese and consumed control diet throughout). Offspring consumed control diet until 11 weeks of age followed by HFS diet until 17 weeks of age. The Obese-OFS + S rats lost weight during the intervention phase whereas the OFS and S treatments attenuated weight gain compared with Obese-HFS (*p* < 0.05). Gestational weight gain was lowest in Obese-OFS + S rats and highest in Obese-HFS rats (*p* < 0.05). Prepregnancy intervention did not affect reproductive parameters but did affect pregnancy outcomes including litter size. Male Obese-OFS offspring had significantly lower percent body fat than Obese-HFS at 17 weeks. Female Obese-S and Obese-OFS offspring had significantly lower fasting glucose at 17 weeks compared with Obese-Control and Obese-HFS. *Clostridium* cluster XI was higher in Obese-HFS and Obese-S dams at birth compared with all other groups. Dams with an adverse pregnancy outcome had significantly lower (*p* = 0.035) *Lactobacillus* spp. compared with dams with normal or small litters. At weaning, male offspring of Obese-HFS had higher levels of *Methanobrevibacter* than all other groups except Obese-S and female Obese-HFS offspring had higher *Enterobacteriaceae* compared with all other groups. At 11 and 17 weeks of age, *Bacteroides*/*Prevotella* spp. was significantly lower in male and female offspring of Obese-HFS dams compared with all other groups except Obese-OFS + S. Modest weight loss induced with a diet-drug combination did not affect maternal fecundity but did have sex-specific effects on offspring adiposity and glycemia that may be linked to changes in offspring microbiota.

## Introduction

Over the past 30 years, worldwide obesity has nearly doubled (1). As a result, a marked increase in preconception body mass index (BMI) in women has also occurred. A growing body of evidence suggests that increasing maternal BMI is associated with a decrease in fecundity (2, 3). Impaired fecundity has also been shown in diet-induced obese mice (4). The Collaborative Perinatal Project (USA) found that the probability of conception is decreased by 18% in women with a BMI ≥ 30 and decreased 8% in women with a BMI between 25 and 29.9 kg/m^2^ compared with women who have a BMI between 18.5 and 24.9 kg/m^2^ (2). Similarly, the Danish National Birth Cohort study found that women with obesity are more likely to undergo infertility treatment than women with normal weight and to take longer than 1 year to conceive (5).

In addition to reduced fecundity, maternal obesity is associated with poor pregnancy outcomes and complications including gestational diabetes, preeclampsia, and macrosomia (6–8). Gestational diabetes and macrosomia are associated with increased risk of childhood obesity, which in turn is associated with obesity in adulthood (8). In sheep, maternal obesity downregulates let-7g microRNA expression in fetal muscle, which may lead to increased intramuscular fat during muscle development (9). Although weight loss in normal weight women before pregnancy has been associated with infants born small for gestational age, weight loss before pregnancy in women with overweight resulted in a trend for reduced maternal risk of gestational diabetes and hypertension, and importantly was not associated with low birth weight in their infants (7). Unfortunately, there is currently a lack of conclusive literature addressing diet-induced weight loss or other interventions before conception in both humans and rodent models (10).

As such, the primary aim of this study was to determine if a combined dietary and pharmacological intervention in the preconception period could promote weight loss in obese Sprague-Dawley rats and thereby improve maternal fecundity, pregnancy outcomes, and metabolic health in the offspring. The dietary intervention was incorporation of the prebiotic oligofructose (OFS) to the diet of the female rats. OFS has been shown to aid in weight loss and glucose control in part *via* increases in the levels of the anorexigenic gut hormone glucagon-like peptide 1 (GLP-1) (11, 12). The pharmacological intervention was the antidiabetic agent sitagliptin, a dipeptidyl peptidase-4 inhibitor used to treat type 2 diabetes by preventing the inactivation of GLP-1 (13). Sitagliptin reduces blood glucose by increasing insulin secretion and suppressing glucagon (14). By combining the actions of OFS to increase endogenous levels of GLP-1 and the actions of sitagliptin to preserve the active form of GLP-1 in circulation, the potential exists for enhanced weight loss and glucose control over either treatment alone.

In addition to its GLP-1 modifying effects, OFS has also been shown to modify the gut microbiota in a manner that reduces inflammation and the propensity for obesity (15–18). The establishment of the gut microbiota appears to be particularly influenced by the early-life environment. The profile of the gut microbiota stabilizes around 3 years of age in humans and is affected to a large extent by maternal factors and the surrounding environment (19, 20). We have recently shown in rats that maternal prebiotic consumption during pregnancy and lactation normalized gestational weight gain, reduced offspring body fat and fasting glucose levels and increased *Bifidobacterium* spp. in both mothers and offspring (21). Whether similar protection is possible with prepregnancy intervention is not known.

Given the increase in rates of obesity in women of child-bearing age and the lack of evaluation of interventions to treat obesity in the preconception period, our objective was to determine if a combined dietary and pharmacological intervention could promote greater weight loss in obese female rats and thereby affect maternal fecundity and pregnancy outcomes and offspring metabolic health. Furthermore, because OFS has proven microbiota modifying effects, we also examined the association between preconception treatment and the gut microbiota profiles in offspring.

## Materials and Methods

### Animals and Experimental Treatments

Ethical approval for the study was granted by the University of Calgary Life and Environmental Science Animal Care Committee and conformed to the *Canadian Council on Animal Care* guidelines. Obesity was induced in 10-week-old female Sprague-Dawley rats (*n* = 120) with a high fat/high sucrose (HFS) diet for 14 weeks as per our previous work (21, 22). The rats with the highest weight gain were then randomized into one of four intervention groups for 8 weeks with AIN-93 control background diet: (1) Obese-Control (obese rats consuming control diet during intervention); (2) Obese-OFS (obese rats consuming 10% OFS diet during intervention); (3) Obese-S (obese rats treated with sitagliptin drug during the intervention); and (4) Obese-OFS + S (obese rats treated with combination treatment). Since the goal of the intervention was weight loss, the treated groups were all switched from the HFS obesity induction diet to a control AIN-93 diet during the intervention and then later in pregnancy. Two groups of reference animals were also included. An untreated obese reference group (Obese-HFS) consumed the HFS diet throughout the study. A lean reference group (Lean-Control) was also included that were never obese and consumed control diet (AIN-93) throughout the study. The standard control diet, the American Institute of Nutrition-93 (AIN-93), and the HFS diet composition have been published previously (23, 24). The HFS diet consisted of (g/100 g) the following: casein (20.0), sucrose (49.9), soybean oil (10.0), lard (10.0), cellulose (5.0), mineral mix (3.5), vitamin mix (1.0), dl-methionine (0.3), choline bitartrate (0.25), and t-butylhydroquinone (0.002) (Dyets Inc., Bethlehem, PA, USA). The OFS diet was prepared by mixing 10 g of OFS (Orafti P95, Beneo, Mannheim, Germany) with 90 g of AIN-93M diet. Sitagliptin (Januvia, Merck Inc.) was administered in the diet as per previous work (25–28) at a dose of 10 mg/kg body weight. Food intake was measured daily, and the concentration of sitagliptin added to the diet adjusted accordingly. At the end of the 8-week intervention, the rats (all 32 weeks of age) were bred with male Sprague-Dawley rats obtained from the University of Calgary Life and Environmental Science Animal Resource Centre (Calgary, AB, USA) and pregnancy confirmed by the presence of a copulation plug.

Dams in the Obese-HFS group (*n* = 12) continued on the HFS diet throughout pregnancy and lactation, while all other dams were fed a control (AIN-93G) diet. If litters exceeded 10 pups at birth, they were culled to 5 male/5 female (where possible) to control for the confounding effects of nutritional differences between a very large litter and smaller ones (29). Pup sex at birth was determined by genital–anal length. Pups were weaned at 3 weeks of age, and one male and one female from each litter were placed on a control (AIN-93G) diet. By selecting one male and one female from each litter, we examined an *n* = 11–13 individual rats per sex that were each derived from a different litter, minimizing the effect of any single dam. Therefore, the total number of offspring is *n* = 11–13 males and *n* = 11–13 females, each from different dams. Offspring body weight was measured weekly on the same day, and food intake was measured for five consecutive days at regular intervals throughout the study. Female offspring were housed at *n* = 3 per treatment per cage until their size precipitated a move to *n* = 2 per cage. Similarly, male offspring were housed at *n* = 2 per treatment per cage until their size required them to be individually housed. Therefore, food intake was calculated based on the total food consumed in the cage divided by the number of rats per cage. At 11 weeks of age, an oral glucose tolerance test (OGTT) was performed to assess glucose tolerance before a metabolic challenge wherein all rats were fed an HFS diet for 6 weeks. A second OGTT was performed at the end of the 6 weeks when offspring were 17 weeks of age. Offspring fecal samples were collected at weaning, 11 weeks and at euthanasia (17 weeks) and stored at −80°C until analysis.

### Maternal Reproductive Parameters

The reproductive parameters measured include the following: (1) fertility, delivery, and pregnancy indexes as per previous work (30); (2) number of live pups at birth (before culling) and pup survival percentage after 2 weeks (31); and (3) sex prevalence (% males/litter). Dams were considered sterile after three unsuccessful breeding attempts (32). Pregnancy outcomes were classified as follows: (1) normal litter (≥10 pups); (2) small litter (<10 pups); (3) adverse (maternal death or ≥half of pups dead within 1 week). We included two groups of reference animals against which the intervention groups could be tested. The inclusion of the Lean-Control group, which had never been obese nor consumed the HFS diet, allowed us to determine if our intervention could mitigate the effects of obesity induction and restore pregnancy outcomes to those of the normal reference rats. The untreated Obese-HFS group provided a reference point for us to determine how beneficial our treatments were compared with rats that remained obese and continued to consume a HFS diet.

### Offspring Metabolic Parameters

Following overnight feed removal, a fasted blood samples was collected from a tail nick and glucose concentrations measured immediately using a One Touch Blood Glucose Meter (BD Biosciences). Immediately following the fasted blood sample, a 2 g/kg glucose load was administered *via* oral gavage, and additional blood glucose measurements made at 15, 30, 60, 90, and 120 min. At the fasting time point, a whole blood sample was collected into tubes containing diprotin-A (0.034 g/L blood: MP Biomedicals, Irvine, CA, USA), Sigma protease inhibitor (1 g/L blood: Sigma Aldrich, Oakville, ON, Canada), and Roche Pefabloc (1 g/L of blood: Roche, Mississauga, ON, Canada) and allowed to clot before centrifugation. Serum concentrations of active GLP-1, total PYY, total GIP, active ghrelin, leptin, and insulin were measured with the Milliplex Rat Gut Hormone Panel kit (Millipore, Billerica, MA, USA). The sensitivity of the kit is as follows (minimum detectable concentration in picograms per milliliter in brackets): GLP-1 (28), GIP (1), ghrelin (2), insulin (28), leptin (27), and PYY (16). The intra-assay variation is <7%, and inter-assay variation is <24%. Insulin resistance was calculated using HOMA-IR with the formula: fasting insulin (μU/ml) × fasting glucose (mmol/l) divided by 22.5. The composite insulin sensitivity index (CISI) was calculated as previously described (17).

### Offspring Body Composition

Offspring were lightly anesthetized with isoflurane and a dual energy X-ray absorptiometry (DXA) scan was performed (Hologic, ODR 4500: Hologic, Bedford, MA, USA). Hologic QDR software for small animals was used to determine lean and fat mass.

### Offspring Gut Microbiota

Fecal samples were collected from offspring at weaning, week 11 and week 17 (euthanasia) for gut microbiota analysis according to our previous work (17, 33). DNA was extracted from the fecal samples using the MP Biomedicals Fast DNA Spin Kit for Feces (MP Biomedicals, Lachine, QC, Canada). Amplification and detection were conducted in 96-well plates with SYBR Green 2× qPCR Master Mix (BioRad). Purified template DNA from reference strains was used to generate standard curves for each primer set using 10-fold serial dilutions of DNA. Standard curves were normalized to copy number of 16S rRNA genes using reference strain genome size and 16S rRNA gene copy number values. Primer sequences for the 10 bacterial groups examined have been previously published (17) and included detection of: (1) *Bifidobacterium* spp. (genus within the Actinobacteria phylum); (2) *Bacteroides*/*Prevotella* spp. (genus within the Bacteroidetes phylum); (3) *Lactobacillus* spp. (genus within the Firmicutes phylum); (4) *Clostridium leptum* (species in the Firmicutes phylum); (5) *Clostridium coccoides* (species in the Firmicutes phylum); (6) *Clostridial* cluster I (group in the Clostridia class of the Firmicutes phylum); (7) *Clostridial* cluster XI (group in the Clostridia class of the Firmicutes phylum); (8) *Roseburia* (genus in the Firmicutes phylum); (9) *Enterobacteriaceae* (family in the Proteobacteria phylum); and (10) *Methanobrevibacter* (species in the Archaea domain). Total bacteria were also measured. Primer selection for qPCR was based on providing broad coverage of the total microbial signal in rats (34). The microbial groups chosen for analysis represent members of the major phyla and classes of the rat gut microbiota, which is similar to human gut microbiota at the phylum level (35, 36). The majority of microbiota in the rat gut belong to the Firmicutes and Bacteroidetes phyla but also include lesser abundance of Actinobacteria (to which bifidobacteria belong) as well as Proteobacteria (*Enterobacteriaceae*).

### Statistical Analysis

All data were analyzed using IBM SPSS Statistics 22 software. The Fisher’s exact test with a Bonferroni adjustment was used to determine if pregnancy outcome differed between maternal treatment groups. Chi square test was used to determine differences between the pregnancy indexes. A one-way ANOVA and Tukey’s *post hoc* multiple comparisons test were used to determine differences in reproductive parameters, weight outcomes, metabolic parameters, and gut microbiota. Repeated measures ANOVA with Tukey’s *post hoc* testing was used to analyze outcomes with multiple time points. Due to the low amount of fecal matter per rat at weaning, samples within sex for a given litter were combined therefore the weaning time point was analyzed separately from the time course analysis at 11 and 17 weeks for offspring microbiota. All outcomes are presented as mean ± SEM. The level of significance was set at *p* ≤ 0.05. Statistical differences are presented using superscripts where mean values with different superscripts are significantly different (*p* < 0.05). For example if three groups have superscripts of a (group 1), b (group 2), and ab (group 3), respectively, then groups 1 and 2 are significantly different because they have unique superscripts whereas group 3 is not significantly different from group 1 or 2 because they share a common superscript.

## Results

### Maternal Weight, Reproductive Parameters, and Pregnancy Outcome

The Obese-OFS + S group lost weight during the treatment phase, and this was statistically different from all other groups (Table 1). There was a significant attenuation in weight gain in the Obese-OFS and Obese-S groups compared with Obese-HFS. Dams in the Obese-HFS group had significantly greater weight gain during the treatment phase compared with dams in the Obese-OFS, Obese-S, and Obese-OFS + S groups. Therefore, body weight was ~11% lower in the Obese-OFS + S rats and ~6% lower in the Obese-OFS and Obese-S rats at mating compared with the Obese-HFS rats. At mating, the Obese-HFS dams weighed significantly more than the Obese-OFS, Obese-OFS + S, and Lean-Control dams (*p* < 0.05). During pregnancy, the Obese-OFS and Obese-OFS + S dams gained the least amount of weight which was significantly different from Lean-Control, Obese-S, and Obese-HFS rats. The Obese-HFS dams gained the most weight during pregnancy (*p* < 0.05). Differences in pup weight were only significant for female pups at birth wherein female pups born to Obese-OFS + S dams were significantly heavier than female pups born to Obese-Control dams (Table 1).

**Table 1:** Body weight outcomes of dams treated with OFS, S, both, or neither and their offspring.

	Lean-Control	Obese-HFS	Obese-Control	Obese-OFS	Obese-S	Obese-OFS + S
**Dams**						
Baseline weight (g)	306 ± 7^a^	339 ± 10^b^	332 ± 7^ab^	331 ± 5^ab^	329 ± 5^ab^	332 ± 5^ab^
Intervention weight change (g)	18.5 ± 4.3^ab^	35.5 ± 6.6^a^	18.9 ± 5.4^ab^	12.1 ± 5.5^b^	13.0 ± 4.0^b^	−6.1 ± 4.4^c^
Weight at breeding (g)	324 ± 6^b^	377 ± 14^a^	348 ± 7^ab^	339 ± 7^b^	347 ± 9^ab^	323 ± 7^b^
Pregnancy weight change (g)	125 ± 7^ac^	131 ± 5^a^	85 ± 11^b^	94 ± 10^bc^	117 ± 7^ac^	90 ± 10^b^
Lactation weight change (g)	15.9 ± 3.7^ab^	0.3 ± 9.2^ab^	10.2 ± 15.5^ab^	10.9 ± 5.1^ab^	−21.9 ± 9^a^	2.3 ± 10.8^ab^

**Offspring**						
Male birth weight (g)	7.2 ± 0.4	7.3 ± 0.4	6.9 ± 0.3	8.3 ± 0.4	7.4 ± 0.4	6.9 ± 0.6
Female birth weight (g)	7.2 ± 0.2^ab^	7.1 ± 0.3^ab^	6.7 ± 0.2^a^	7.7 ± 0.3^ab^	7.3 ± 0.3^ab^	8.0 ± 0.3^b^
Male weaning weight (g)	60 ± 4.9	60.9 ± 5.7	51.6 ± 7	51.8 ± 5.4	58.7 ± 5.2	53.4 ± 5.2
Female weaning weight (g)	56 ± 5	45.8 ± 7.2	38.5 ± 5.5	50.3 ± 5.5	55.3 ± 5.3	51.7 ± 5.3

*Values are presented as mean ± SEM (*n* = 11–13 per group)*.

*HFS, high fat/sucrose; OFS, oligofructose; S, sitagliptin*.

Obese in the group name indicates diet-induced obese rats that underwent the respective prepregnancy interventions, except Obese-HFS that remained untreated and consumed HFS diet throughout the study. Lean in the group name indicates a lean reference group that never underwent the obesity induction period and consumed control AIN-93 diet throughout the study. Baseline weight refers to body weight after the obesity induction period and just before starting the diet and drug preconception treatments. Mean values with different superscripts are significantly different between treatment groups (*p* < 0.05). For example, a group with “^a^” is significantly different from a group with “^b^” but not from a group with “^ab^.”

No statistically significant differences were found across the maternal treatment groups for the reproductive parameters examined, including fertility, time to successful mating, delivery and pregnancy index, live births, pup survival and sex prevalence (Table 2). Maternal treatment did, however, have a significant effect on pregnancy outcome (*p* = 0.017; Table 3). Obese-S dams were significantly more likely to have a smaller litter size than Obese-Control dams. Although not significant, none of the Obese-HFS dams had a normal litter and across all treatments, Obese-HFS dams had the greatest number of adverse outcomes (Table 3).

**Table 2:** Reproductive parameters of dams treated prepregnancy with OFS, S, both, or neither.

	Lean-Control	Obese-HFS	Obese-Control	Obese-OFS	Obese-S	Obese-OFS + S
Mated rats	13/13 (100%)	12/12 (100%)	13/13 (100%)	12/12 (100%)	13/13 (100%)	12/12 (100%)
Fertility index	13/13 (100%)	10/12 (83.3%)	13/13 (100%)	10/12 (83.3%)	13/13 (100%)	12/12 (100%)
Delivery index	10/13 (76.9%)	10/10 (100%)	11/13 (84.6%)	9/10 (90.0%)	11/13 (84.6%)	9/12 (75.0%)
Pregnancy index	9/13 (69.2%)	8/10 (80.0%)	11/13 (84.6%)	8/10 (80.0%)	10/13 (76.9%)	8/12 (66.7%)
Litter size (# live at birth)	10.3 ± 1.5	7.14 ± 1.3	8.5 ± 1.5	11.1 ± 1.2	8.33 ± 0.8	6.57 ± 1.1
Pup survival (% live at 2 weeks)	82.5	63.7	57.4	64.4	82.7	81.0
Sex prevalence (% males)	38.1	51.6	51.6	40.4	50.4	51.1

*Values are presented as mean ± SEM (*n* = 11–13 per group)*.

*HFS, high fat/sucrose; OFS, oligofructose; S, sitagliptin*.

*Obese in the group name indicates diet-induced obese rats that underwent the respective prepregnancy interventions, except Obese-HFS that remained untreated and consumed HFS diet throughout the study. Lean in the group name indicates a lean reference group that never underwent the obesity induction period and consumed control AIN-93 diet throughout the study. No statistically significant differences exist between maternal treatment groups (*p* ≥ 0.05)*.

**Table 3:** Percentage of pregnancies with normal, small, or adverse litters in dams treated with OFS, S, both, or neither.

	Lean-Control	Obese-HFS	Obese-Control	Obese-OFS	Obese-S	Obese-OFS + S
Normal litter	50	0	50	44	18	0
Small litter	20^ab^	45^ab^	0^a^	12^ab^	64^b^	67^ab^
Adverse	30	55	50	44	18	33

*Values are presented as mean percentage of all pregnancies resulting in normal, small, or adverse litter status (*n* = 9 for Obese-OFS and Obese-OFS + S; *n* = 10 for Lean-Control and Obese-Control; *n* = 11 for Obese-HFS and Obese-S)*.

*HFS, high fat/sucrose; OFS, oligofructose; S, sitagliptin*.

*Obese in the group name indicates diet-induced obese rats that underwent the respective prepregnancy interventions, except Obese-HFS that remained untreated and consumed HFS diet throughout the study. Lean in the group name indicates a lean reference group that never underwent the obesity induction period and consumed control AIN-93 diet throughout the study. Mean values with different superscripts are significantly different between treatment groups (*p* < 0.05)*.

### Maternal Gut Microbiota at Birth

**Figure 1:**
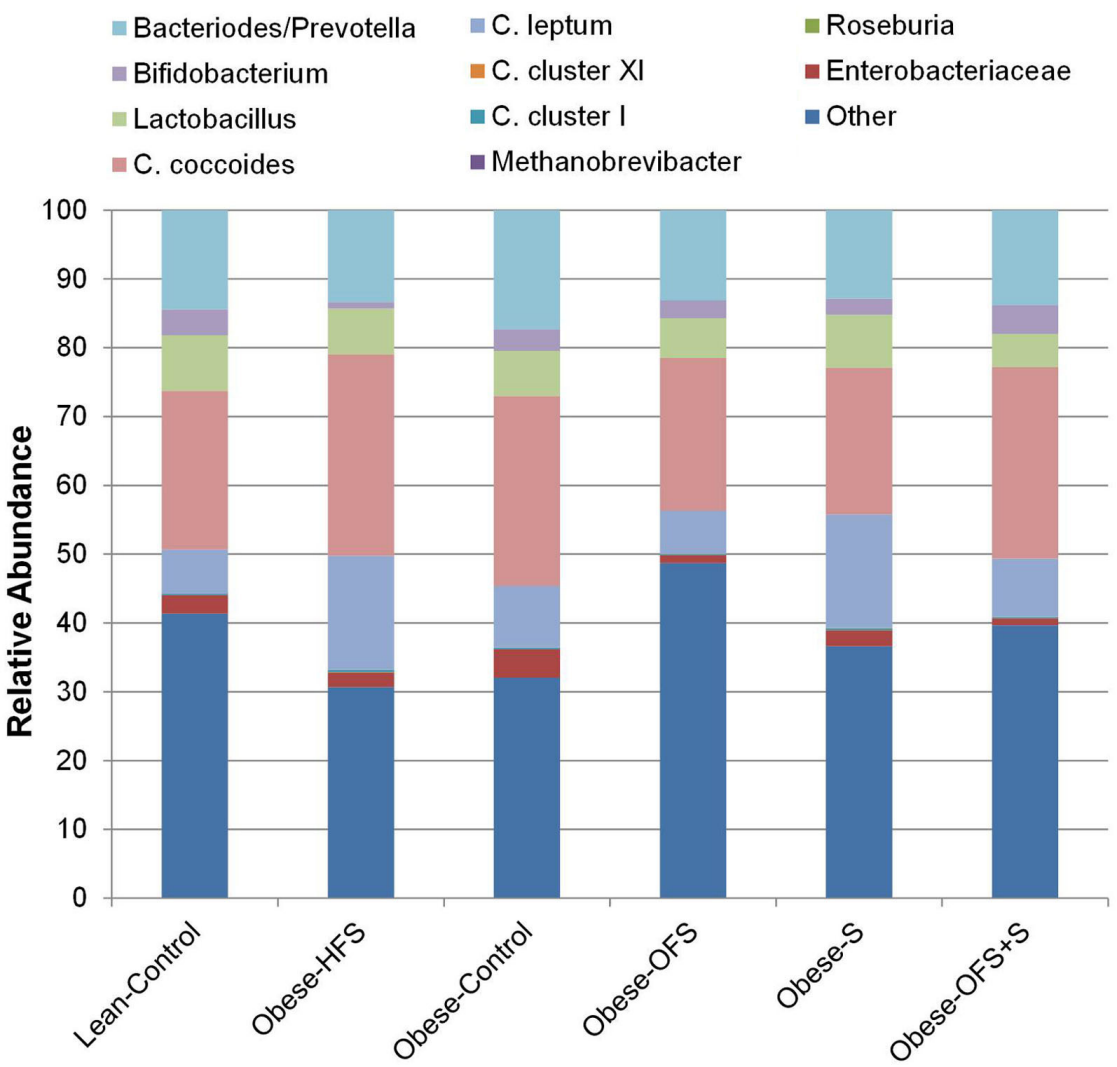
Maternal gut microbiota at birth in dams treated prepregnancy with oligofructose (OFS), sitagliptin, both, or neither. Values are mean relative abundance of fecal microbiota (*n* = 9–11). Microbial abundance was measured as 16S rRNA gene copies per 20 ng DNA and reported here as relative abundance (%) of bacterial taxa per total bacteria.

Maternal gut microbiota composition at birth showed a significant treatment effect for *Clostridium* cluster XI (*p* = 0.0001) wherein abundance was significantly higher in Obese-HFS and Obese-S dams compared with all other groups (*p* < 0.02; Figure 1). *Clostridium* cluster I was significantly higher in Obese-HFS compared with all other groups except Obese-S (*p* < 0.027). There was a trend (*p* = 0.063) for *C. leptum* to be higher in Obese-HFS and Obese-S compared with the other groups. There were two significant differences detected in gut microbiota when assessed according to pregnancy outcome. Dams that had an adverse pregnancy outcome, had significantly lower (*p* = 0.042) *Lactobacillus* spp. (4.5 ± 0.3% relative abundance) compared to dams with normal (7.5 ± 0.8% relative abundance) or small litters (6.9 ± 0.8% relative abundance). Dams with small litters had significantly (*p* = 0.038) higher *C*. *leptum* (14.5 ± 2.7% relative abundance) compared with adverse (8.3 ± 1.2% relative abundance) and normal (7.2 ± 1.8% relative abundance) litters.

### Offspring Body Composition

**Figure 2:**
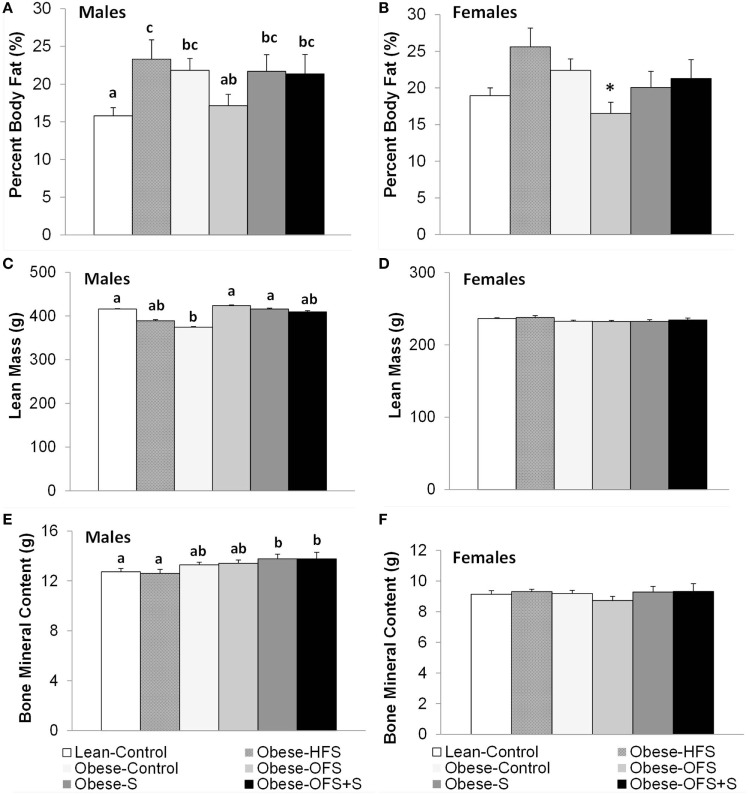
Body composition at 17 weeks of age of male **(A,C,E)** and female **(B,D,F)** offspring of dams treated with oligofructose (OFS), sitagliptin, both, or neither. Values are mean ± SEM (*n* = 8–11). Within a sex, groups without a common superscript (^a,b^) are significantly different (*p* < 0.05). *Represents a trend (*p* = 0.068) for Obese-OFS to be lower than Obese-HFS and Obese-Control within females.

Percent body fat was significantly lower in male Lean-Control offspring compared with all other groups (*p* < 0.05) except Obese-OFS (Figure 2). Obese-OFS male offspring had lower percent body fat compared with Obese-HFS (*p* = 0.03). In female offspring, there was a trend (*p* = 0.068) for Obese-OFS to have lower percent body fat compared with Obese-HFS and Obese-Control offspring. Lean mass in male offspring was significantly higher in Lean-Control (*p* = 0.028), Obese-OFS (*p* = 0.019) and Obese-S (*p* = 0.033) compared with Obese-Control (Figure 2). Bone mineral content in male offspring was significantly higher in Obese-S (*p* < 0.019) and Obese-OFS + S (*p* < 0.03) compared with Lean-Control and Obese-HFS. There were no differences in naso-anal length among groups in male or female offspring (*p* > 0.05).

### Offspring Glucose Tolerance and Fasting Satiety Hormones

**Figure 3:**
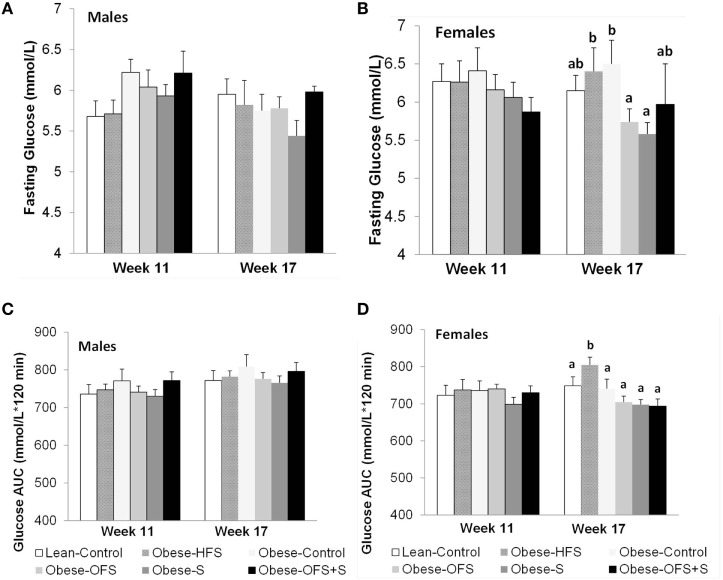
Fasting blood glucose and glucose AUC at 11 and 17 weeks of age of male **(A,C)** and female **(B,D)** offspring of dams treated with oligofructose (OFS), sitagliptin, both, or neither. Values are mean ± SEM (*n* = 8–11). Within a sex, groups without a common superscript (^a,b^) are significantly different (*p* < 0.05).

**Figure 4:**
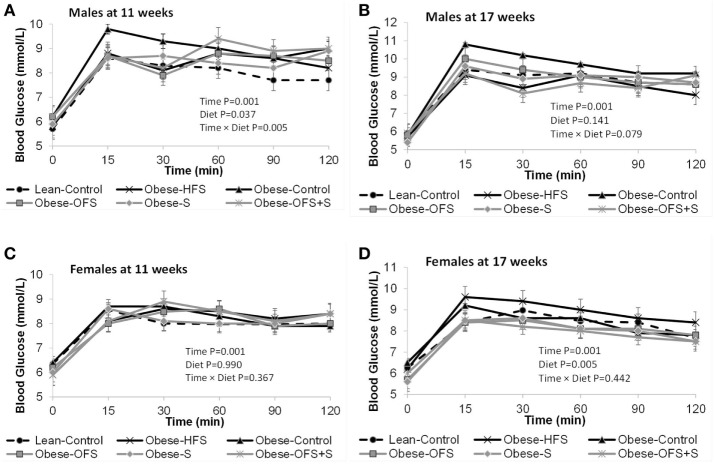
Blood glucose concentrations during the oral glucose tolerance test at 11 and 17 weeks of age in male **(A,B)** and female **(C,D)** offspring of dams treated with oligofructose (OFS), sitagliptin, both, or neither. Values are mean ± SEM (*n* = 8–11).

There were no changes in fasting blood glucose or glucose AUC at 11 weeks of age in males or females (Figure 3). At 17 weeks, however, female offspring of Obese-OFS and Obese-S dams had significantly lower fasting blood glucose compared with Obese-HFS and Obese-Control offspring (*p* < 0.05). Glucose AUC during the OGTT was significantly higher in Obese-HFS female offspring compared with all other groups at 17 weeks (*p* < 0.05). As expected, during the OGTT, there was a significant effect of time for the glucose curves at both 11 and 17 weeks for male and female offspring (*p* = 0.001) (Figure 4). There was a significant interaction between time × diet (*p* = 0.005) observed in the males during the OGTT at 11 weeks wherein Obese-Control was higher than Obese-OFS (*p* = 0.004) at 30 min, Obese-OFS + S was higher than Lean-Control at 90 min and Obese-Control was higher than Lean-Control at 120 min. There was a significant effect of diet (*p* = 0.005) in the females at 17 weeks with Obese-HFS being higher than all other groups except Obese-Control.

Fasting ghrelin was significantly lower in male Obese-OFS, Obese-S, and Obese-HFS compared with Lean-Control (*p* < 0.04) (Table 4). In females, Lean-Control had significantly higher fasting ghrelin compared with all other groups (*p* < 0.036). Although HOMA-IR tended to be lower and CISI tended to be higher (*p* = 0.10) in male Obese-S, this was not significantly different (Table 4).

**Table 4:** Fasting serum satiety hormones, HOMA-IR, composite insulin sensitivity index (CISI) at 17 weeks and energy intake in offspring (weeks 4, 10, and 16).

	Sex	Lean-Control	Obese-HFS	Obese-Control	Obese-OFS	Obese-S	Obese-OFS + S
Insulin, pg/ml	M	1,406 ± 292	1,207 ± 217	1,613 ± 246	1,597 ± 127	1,274 ± 338	1,783 ± 389
	F	895 ± 185	788 ± 94.4	1,174 ± 321	1,149 ± 263	1,290 ± 488	1,134 ± 350
Leptin, pg/ml	M	4,866 ± 1,106	4,174 ± 953	3,251 ± 770	5,748 ± 1,015	2,183 ± 492	5,464 ± 1,303
	F	5,779 ± 843	3,706 ± 665	3,286 ± 954	5,698 ± 746	4,854 ± 974	4,669 ± 817
Ghrelin, pg/ml	M	178.2 ± 30.0^a^	63.1 ± 21.4^b^	145.9 ± 35.5^ab^	46.9 ± 12.5^b^	55.8 ± 12.8^b^	131.7 ± 35.7^ab^
	F	299 ± 34.5^a^	194 ± 31.2^b^	188 ± 47.3^b^	117 ± 16.1^b^	200.8 ± 29.0^b^	186 ± 25.8^b^
GIP, pg/ml	M	106.8 ± 31.3	42.7 ± 14.4	41.3 ± 7.8	58.0 ± 16.2	37.9 ± 6.3	55.8 ± 11.9
	F	124 ± 30.2	46 ± 13.1	96.8 ± 30.2	112 ± 38.6	86.5 ± 23.2	79.7 ± 15.7
PYY, pg/ml	M	29.5 ± 2.8	28.1 ± 5.8	26.8 ± 6.1	26.1 ± 6.0	25.8 ± 4.4	39.4 ± 11.9
	F	42.1 ± 23	25.0 ± 7.0	41.4 ± 6.8	42.1 ± 9.0	45.7 ± 11.1	37.3 ± 8.4
Glucagon-like peptide 1, pg/ml	M	11.4 ± 3.0	10.5 ± 3.3	11.5 ± 2.9	9.8 ± 2.5	10.3 ± 2.5	12.4 ± 2.7
	F	20.1 ± 18	12.4 ± 9.0	6.1 ± 3.3	10.4 ± 5.0	5.4 ± 3.0	9.6 ± 4.2
HOMA-IR	M	8.9 ± 2.0	7.2 ± 1.5	11.1 ± 1.5	9.8 ± 0.8	5.4 ± 1.1	8.9 ± 2.2
	F	6.2 ± 1.4	5.7 ± 0.9	7.9 ± 2.4	7.1 ± 1.7	7.8 ± 3.2	7.3 ± 2.2
CISI	M	0.49 ± 0.08	0.52 ± 0.06	0.33 ± 0.05	0.36 ± 0.02	0.73 ± 0.22	0.46 ± 0.09
	F	0.78 ± 0.14	0.73 ± 0.08	0.68 ± 0.12	0.75 ± 0.15	0.73 ± 0.10	0.75 ± 0.09
Average energy intake (week 4), kcal/d	M	42.2 ± 3.1	54.5 ± 10.4	44.3 ± 3.7	49.5 ± 14.1	38.9 ± 6.9	41.4 ± 6.2
	F	26.3 ± 3.0	45.5 ± 10.4	26.1 ± 1.1	25.2 ± 1.8	26.6 ± 1.8	35.8 ± 2.4
Average energy intake (week 10), kcal/d	M	63.5 ± 4.3	82.0 ± 8.7	72.9 ± 2.4	77.9 ± 3.7	77.8 ± 4.8	70.0 ± 8.5
	F	43.7 ± 1.1^a^	72.0 ± 1.3^b^	56.2 ± 4.8^ab^	57.1 ± 4.8^ab^	44.9 ± 2.5^a^	59.0 ± 8.9^ab^
Average energy intake (week 16), kcal/d	M	73.8 ± 7.0	88.8 ± 14.8	80.1 ± 4.9	78.2 ± 3.8	86.7 ± 8.8	80.3 ± 6.0
	F	50.2 ± 4.0^a^	79.6 ± 1.5^b^	73.5 ± 8.7^ab^	77.3 ± 4.4^ab^	60.0 ± 3.0^ab^	81.3 ± 2.2^b^

*Values are means ± SEM (*n* = 8–11 per group). Serum satiety hormones, HOMA-IR, and CISI were measured at 17 weeks of age. Energy intake was measured for five consecutive days in the week indicated and averaged to kcal/day per rat across an *n* = 2 male or *n* = 3 female rats per cage*.

*^ab^Values without common superscript are significantly different within a sex (*p* < 0.05)*.

*Txt, treatment*.

Energy intake was measured during weeks 4, 7, 10, 13, and 16. In females, there were significant differences detected at weeks 10 and 16 with Obese-HFS consuming more energy than Lean-Control (*p* = 0.021) and more than Obese-S (*p* = 0.008) at 10 weeks (Table 4). Lean-Control consumed less energy compared with Obese-HFS (*p* = 0.034) and Obese-OFS + S (*p* = 0.011) at week 16. There were no significant differences in energy intake in males.

### Offspring Gut Microbiota

There was a significant effect of sex for gut microbiota, therefore males and females were analyzed separately. At weaning, there was a significant difference in *Methanobrevibacter* spp. (*p* = 0.02) with male offspring of Obese-HFS having higher levels (0.0081 ± 0.001% relative abundance) compared with Lean-Control (0.0024 ± 0.001%), Obese-Control (0.0037 ± 0.001%), Obese-OFS (0.0038 ± 0.001%), and Obese-OFS + S (0.0037 ± 0.001%) but not Obese-S (0.00550 ± 001%). In female offspring at weaning, there was a significant difference (*p* = 0.018) in *Enterobacteriaceae* with offspring of Obese-HFS dams having higher levels (1.22 ± 0.49% relative abundance) compared with all other groups: Obese-OFS (0.120 ± 05%), Obese-S (0.020 ± 01%), Obese-OFS + S (0.20 ± 0.09%), Obese-HFS (0.26 ± 0.06%), and Lean-Control (0.45 ± 0.15%).

At 11 and 17 weeks of age in male offspring, there was a significant effect of time (*p* < 0.01) for *Bacteroides*/*Prevotella* spp., *Bifidobacterium* spp., *Methanobrevibacter* spp., *C. leptum*, and *C*. Cluster I (Table 5). There was a significant effect of treatment (*p* = 0.039) on *Bacteroides*/*Prevotella* spp. wherein male offspring of Obese-HFS dams had lower abundance than all other groups except Obese-OFS + S at 11 weeks. For *C*. cluster I, Obese-HFS offspring had significantly higher abundance compared with all other groups at 11 weeks. The interaction between time and treatment affected *Lactobacillus* spp. (*p* = 0.031) wherein male offspring of Obese-HFS dams had lower relative abundance than offspring from Lean-Control at 11 weeks of age. By 17 weeks of age, all groups were significantly lower than their week 11 abundance. *Roseburia* was also affected by the interaction of time and treatment (*p* = 0.025) wherein male offspring of Lean-Control had significantly higher abundance than Obese-OFS + S at 11 weeks and by 17 weeks of age, Lean-Control was higher than Obese-OFS, Obese-S and Obese-OFS + S.

**Table 5:** Male offspring gut microbiota at 11 and 17 weeks of age.

	Week	Lean-Control	Obese-HFS	Obese-Control	Obese-OFS	Obese-S	Obese-OFS + S	Time	Txt	Time × Txt
*Bacteroides*/*Prevotella*	11	13.1 ± 2.4	7.0 ± 0.7	15.2 ± 2.1	18.8 ± 2.5	17.9 ± 1.9	12.3 ± 2.1	0.001	0.039	0.579
	17	8.5 ± 0.9	8.1 ± 1.2	8.8 ± 1.4	13.3 ± 3.9	12.1 ± 1.4	8.6 ± 1.0			
*Bifidobacterium*	11	2.8 ± 1.5	0.5 ± 0.2	0.7 ± 0.2	1.1 ± 0.3	1.0 ± 0.4	1.9 ± 0.7	0.0001	0.308	0.296
	17	0.8 ± 0.1	0.7 ± 0.1	0.8 ± 0.2	0.7 ± 0.1	0.9 ± 0.1	0.7 ± 0.1			
*Methanobrevibacter*	11	0.02 ± 0.002	0.02 ± 0.004	0.02 ± 0.003	0.02 ± 0.001	0.02 ± 0.003	0.02 ± 0.004	0.001	0.949	0.850
	17	0.08 ± 0.01	0.07 ± 0.01	0.08 ± 0.02	0.07 ± 0.01	0.09 ± 0.01	0.07 ± 0.01			
*Enterobacteriaceae*	11	0.10 ± 0.05	0.06 ± 0.02	0.04 ± 0.01	0.04 ± 0.01	0.08 ± 0.02	0.11 ± 0.03	0.393	0.555	0.391
	17	0.06 ± 0.01	0.26 ± 0.20	0.15 ± 0.04	0.04 ± 0.01	0.08 ± 0.02	0.05 ± 0.02			
*Lactobacillus*	11	16.7 ± 4.3^a^	6.4 ± 1.7^b^	7.0 ± 1.6^ab^	10.0 ± 1.7^ab^	10.5 ± 2.7^ab^	10.5 ± 1.9^ab^	0.001	0.065	0.031
	17	2.3 ± 0.7^c^	4.3 ± 1.4^c^	3.5 ± 1.5^c^	2.1 ± 0.6^c^	1.6 ± 0.7^c^	1.9 ± 0.7^c^			
*Clostridium coccoides*	11	26.5 ± 6.6	15.0 ± 3.3	23.6 ± 4.0	26.1 ± 4.96	14.1 ± 1.6	19.2 ± 2.8	0.257	0.753	0.065
	17	18.8 ± 1.8	25.5 ± 4.0	2.3 ± 3.1	24.4 ± 3.6	26.7 ± 2.7	25.1 ± 3.5			
*Clostridium leptum*	11	5.9 ± 2.5	8.3 ± 2.4	2.1 ± 0.9	2.1 ± 0.4	2.2 ± 0.5	2.1 ± 0.9	0.001	0.403	0.118
	17	10.8 ± 2.0	12.6 ± 2.6	8.5 ± 1.8	16.4 ± 4.7	16.6 ± 2.8	10.1 ± 1.9			
*Clostridium* cluster XI	11	0.12 ± 0.04	0.19 ± 0.03	0.11 ± 0.02	0.13 ± 0.02	0.14 ± 0.02	0.20 ± 0.06	0.291	0.563	0.472
	17	0.30 ± 0.15	0.50 ± 0.06	0.99 ± 0.79	0.10 ± 0.04	0.09 ± 0.03	0.07 ± 0.01			
*Clostridium* cluster I	11	0.66 ± 0.16	1.22 ± 0.22	0.53 ± 0.09	0.68 ± 0.15	0.46 ± 0.07	0.72 ± 0.21	0.016	0.004	0.110
	17	0.57 ± 0.19	0.58 ± 0.08	0.51 ± 0.13	0.34 ± 0.09	0.30 ± 0.04	0.41 ± 0.07			
*Roseburia*	11	4.9 ± 1.2^a^	2.2 ± 0.8^ab^	2.5 ± 0.7^ab^	2.1 ± 0.7^ab^	1.8 ± 0.7^ab^	1.2 ± 0.1^b^	0.001	0.019	0.025
	17	0.08 ± 0.02^c^	0.03 ± 0.01^cd^	0.03 ± 0.02^cd^	0.01 ± 0.003^d^	0.02 ± 0.005^d^	0.01 ± 0.002^d^			

*Values are mean ± SEM (*n* = 8–11) expressed as the relative abundance (%) of bacterial taxa per total bacteria (16S rRNA gene copies of microbial group/total bacteria 16S rRNA gene copies). Week 11 represents values just before starting the high fat/sucrose (HFS) diet whereas week 17 represents values after 6 weeks of HFS diet*.

*Txt, treatment*.

*When a significant interaction effect was found, significant differences between groups were identified with Tukey’s *post hoc* tests. Values that do not share a common superscript are significantly different (*p* < 0.05)*.

In female offspring, there was a significant effect of time (*p* < 0.024) for *Bifidobacterium* spp., *Methanobrevibacter* spp., *Lactobacillus* spp., *C. leptum, C*. cluster XI, and *C*. cluster I (Table 6). There was a significant effect of treatment (*p* = 0.003) for *Bacteroides*/*Prevotella* spp. wherein female offspring of Obese-HFS dams had significantly lower abundance compared with all other groups except Obese-OFS + S.

**Table 6:** Female offspring gut microbiota at 11 and 17 weeks.

	Week	Lean-Control	Obese-HFS	Obese-Control	Obese-OFS	Obese-S	Obese-OFS + S	Time	Txt	Time × Txt
*Bacteroides*/*Prevotella*	11	12.9 ± 1.6	7.5 ± 0.6	13.2 ± 1.1	12.4 ± 2.6	16.7 ± 1.8	10.5 ± 2.0	0.984	0.003	0.911
	17	12.3 ± 1.1	9.0 ± 1.1	12.7 ± 1.8	10.8 ± 1.7	14.7 ± 2.2	11.8 ± 3.0			
*Bifidobacterium*	11	2.4 ± 0.8	1.9 ± 0.6	1.8 ± 0.8	1.1 ± 0.2	1.7 ± 0.3	2.9 ± 0.9	0.001	0.354	0.248
	17	0.59 ± 0.23	0.09 ± 0.03	0.40 ± 0.10	0.13 ± 0.05	0.10 ± 0.02	0.19 ± 0.04			
*Methanobrevibacter*	11	0.07 ± 0.01	0.10 ± 0.02	0.09 ± 0.01	0.08 ± 0.02	0.06 ± 0.01	0.07 ± 0.01	0.024	0.989	0.221
	17	0.07 ± 0.01	0.05 ± 0.01	0.05 ± 0.01	0.05 ± 0.01	0.07 ± 0.01	0.07 ± 0.01			
*Enterobacteriaceae*	11	0.36 ± 0.22	0.10 ± 0.04	0.02 ± 0.01	0.04 ± 0.01	0.02 ± 0.003	0.09 ± 0.02	0.863	0.080	0.969
	17	0.23 ± 0.21	0.04 ± 0.01	0.09 ± 0.03	0.05 ± 0.02	0.05 ± 0.02	0.07 ± 0.02			
*Lactobacillus*	11	7.2 ± 2.2	8.4 ± 2.1	7.8 ± 2.6	4.1 ± 1.5	5.8 ± 0.7	5.9 ± 1.7	0.001	0.530	0.330
	17	3.5 ± 2.2	1.1 ± 0.3	2.6 ± 1.1	1.3 ± 0.4	1.4 ± 0.8	1.7 ± 0.6			
*Clostridium coccoides*	11	26.6 ± 3.5	23.0 ± 2.7	22.3 ± 3.2	27.2 ± 4.1	18.5 ± 2.5	22.6 ± 3.3	0.496	0.316	0.182
	17	19.4 ± 2.4	32.0 ± 5.3	23.1 ± 4.1	24.7 ± 2.9	22.7 ± 1.9	28.9 ± 3.3			
*Clostridium leptum*	11	10.6 ± 2.0	6.3 ± 1.1	6.4 ± 1.2	7.4 ± 1.7	9.8 ± 1.5	7.5 ± 0.9	0.001	0.218	0.781
	17	16.0 ± 2.5	12.2 ± 2.4	9.4 ± 2.7	16.3 ± 3.1	11.7 ± 2.1	13.0 ± 3.2			
*Clostridium* cluster XI	11	0.03 ± 0.005	0.07 ± 0.02	0.03 ± 0.004	0.03 ± 0.004	0.02 ± 0.004	0.05 ± 0.02	0.003	0.442	0.658
	17	0.16 ± 0.05	0.21 ± 0.09	0.19 ± 0.07	0.11 ± 0.06	0.04 ± 0.02	0.17 ± 0.06			
*Clostridium* cluster I	11	0.54 ± 0.09	0.81 ± 0.18	0.71 ± 0.10	0.46 ± 0.08	0.75 ± 0.04	0.73 ± 0.18	0.001	0.417	0.714
	17	0.03 ± 0.01	0.06 ± 0.05	0.05 ± 0.01	0.01 ± 0.002	0.03 ± 0.01	0.05 ± 0.02			
*Roseburia*	11	0.02 ± 0.01	0.02 ± 0.01	0.01 ± 0.002	0.01 ± 0.003	0.01 ± 0.004	0.01 ± 0.004	0.323	0.285	0.198
	17	0.02 ± 0.01	0.01 ± 0.005	0.03 ± 0.02	0.01 ± 0.004	0.01 ± 0.004	0.01 ± 0.004			

*Values are mean ± SEM (*n* = 8–11) expressed as the relative abundance (%) of bacterial taxa per total bacteria (16S rRNA gene copies of microbial group/total bacteria 16S rRNA gene copies). Week 11 represents values just before starting the high fat/sucrose (HFS) diet whereas week 17 represents values after 6 weeks of HFS diet*.

*Txt, treatment*.

## Discussion

Obesity in women of child-bearing age shows a strong association with female infertility (2, 5). Subfecundity, or reduced capacity to conceive, in women with obesity is thought to result in part from hormonal disturbances and insulin resistance (37, 38). Reducing body weight with a very low calorie diet in patients with infertility and obesity resulted in an increase in insulin sensitivity that was inversely related to a decrease in luteinizing hormone (39). Given that excessive body fat and insulin resistance play a large role in obesity-related infertility, we tested whether a prepregnancy treatment that combined the effects of OFS and sitagliptin would enhance weight loss before conception and thereby improve maternal fecundity and offspring outcomes. This study found that despite a significant reduction (11%) in body weight in the combined treatment group just before conception, there were no resulting reproductive benefits. It also appears that pregnancy outcome, independent of maternal weight change, affects offspring birth weight and weight at the time of weaning. In addition, changes in maternal microbiota, particularly in untreated Obese-HFS dams, may impact the heritability of offspring microbiota and long-term metabolic health.

Supplementation with OFS is associated with a reduction in body fat in rodents and in adults and children with overweight or obesity (12, 16, 21, 40). As hypothesized, the actions of OFS and sitagliptin resulted in greater weight loss than either treatment alone in obese female rats in the prepregnancy period. Although the treatments administered as monotherapy did not induce weight loss they did attenuate weight gain which is relevant because rats continue to gain weight over the majority of their life span (41). Despite the weight loss induced by the OFS + S treatment, there were no associated improvements in fecundity or pregnancy outcome. One explanation for this finding could be that despite significant weight loss that resulted in a body weight that was 11% lower at mating than the Obese-HFS rats, the dams did not lose sufficient weight (and potentially more important fat mass) to result in measureable changes in fecundity. Surgical weight loss has shown positive effects on obesity-related infertility. One study showed that 62.7% of infertile women who underwent bariatric surgery were able to conceive after the surgical intervention (38). Specifically, that study showed that a weight loss of BMI > 5 kg/m^2^ was one of the best predictors of becoming pregnant following surgery (38). The results of our study may more closely resemble Chavarro et al., where short-term weight loss did not result in an improvement in fertility treatment outcomes (42). In terms of pregnancy outcomes, a recent Canadian cohort study showed that a 10% difference in prepregnancy BMI-affected risk for preeclampsia, gestational diabetes, indicated preterm delivery, macrosomia, and still birth whereas larger differences in BMI were necessary to see lowered risk of cesarean delivery, in-hospital newborn mortality, shoulder dystocia, and neonatal intensive care unit stays (43).

Many negative health consequences have been associated with low birth weight including enhanced risk of obesity, diabetes, and other metabolic-related diseases later in life (8, 44). Similar to Diouf et al. (7), weight loss before pregnancy in obese rats did not result in low birth weight in our offspring. Instead, pregnancy outcome appears to be a better predictor of offspring weight at birth and weaning in our study than maternal treatment. Pups born to dams with adverse litter outcomes had significantly lower birth weight than pups born in small or normal litters. The main causes of adverse pregnancy outcomes were pup death and maternal death shortly after parturition due to pregnancy toxemia, which matches the current literature where hypertensive disorders are associated with low birth weight irrespective of maternal prepregnancy BMI (45, 46). The results did not show a direct association between maternal treatment and low birth weight or between maternal treatment and adverse pregnancy outcomes. Factors independent of maternal weight status could have affected pregnancy outcome and low birth weight status. Although not statistically significant, pup survival at 2 weeks of age was lower in the obese dams than the Lean-Control dams, a finding that has been shown previously (47). Higher peripartum pup mortality in obesity has been linked in part to impaired lactogenesis (47, 48). Switching obese pregnant rats at parturition from a high-fat diet to a low-fat diet improved milk production and pup growth (48). We did not measure milk production in our study and therefore it remains to be elucidated whether a preconception intervention could improve lactogenesis in obese rats.

We have previously shown that parturition is characterized by higher *Enterobacteriaceae* and *C. coccoides* and lower *Bacteroides*/*Prevotella, Lactobacillus, C. leptum*, and C*lostridial* cluster I compared with lactation in Wistar rat dams (49). The changes in microbiota triggered by pregnancy and lactation appear to be greater than those induced by diet because in contrast to the numerous microbe changes listed above, only *Bifidobacterium* spp. and *C. coccoides* were altered in that study when the pregnant dams were fed a high prebiotic fiber diet (49). Similarly, we did not detect marked changes due to diet or drug intervention in the microbial profile between our maternal treatment groups at parturition, except for increased Cluster XI and I abundance in Obese-S. Perhaps more interesting is the decreased abundance of *Lactobacillus* detected in dams with adverse pregnancy outcomes. Reduced abundance of vaginal *Lactobacillus* and higher bacterial diversity has been associated with preterm delivery (50–53). *Bifidobacterium* and *Lactobacillus* strains are the most common probiotics, and their use has been associated with several benefits including reduction in upper respiratory tract infections in childhood (54). In pregnant women, a probiotic supplement containing *Lactobacillus acidophilus, Lactobacillus casei*, and *Bifidobacterium bifidum* decreased fasting glucose, C-reactive protein, and plasma malondialdehyde, a marker of oxidative stress but did not affect pregnancy outcome (55).

In the offspring, the obesity status of the dams affected offspring gut microbiota more so than the maternal preconception diet and drug interventions. Both male and female offspring of untreated Obese-HFS dams had reduced abundance of *Bacteroides*/*Prevotella* spp. compared with the Lean-Control and the treated Obese dams (including the Obese-Control dams that did not receive OFS or sitagliptin prepregnancy and their only “treatment” was consuming control AIN-93G diet during pregnancy and lactation compared with Obese-HFS that consumed HFS continuously). *Bacteroides*/*Prevotella* has been shown to be lower in subjects with obesity compared with healthy weight subjects and it is negatively associated with BMI and body fat mass (56). *Lactobacillus* was also compromised in male offspring of untreated Obese-HFS dams. Their abundance at 11 weeks was less than half of that seen in offspring of Lean-Control dams. *Lactobacillus* is recognized for its health promoting properties (17, 57) and has been shown in Sprague-Dawley and Wistar rats to be decreased in response to a high-fat diet (58). Interestingly, preconception treatment with OFS, S, or both showed a tendency to correct some of this dysbiosis with increased *Lactobacillus* spp., however, it remained lower than Lean-Control. Restoration of *Lactobacillus* may be important given evidence that a *L. casei* strain Shirota-containing beverage contributed to weight loss and improved lipid metabolism in children with obesity (59). Although we did not detect significant effects of diet or drug intervention on *Bacteroides*/*Prevotella* and *Lactobacillus* in the dams (but did according to pregnancy outcome), there is in fact a complex relationship between maternal and offspring microbial profile. One study looking at the microbial relationship between mother and infant found that the highest dissimilarity between maternal and offspring microbiota occurred between late pregnancy and 3-day-old newborns (60). Koren et al. showed that maternal microbiota is markedly altered between the first trimester and the third trimester of pregnancy and that offspring microbial profile does not start to resemble the maternal first trimester microbial profile in humans until 4 years of age (61). Interestingly although it was not significant, the untreated Obese-HFS dams had the lowest abundance of *Bacteroides*/*Prevotella* spp. and *Lactobacillus* spp. at birth compared with the other groups and these patterns would be captured in the overall community structure of maternal gut microbiota involved in vertical transmission to the pups at birth (62).

This study is not without limitations. The age of the dams at the time of breeding (32 weeks of age) may have precluded an improvement in fecundity that was large enough to detect. However, since all of the dams were roughly the same age during breeding, any reductions in fecundity would have been systematic across all of the treatment groups. Given the evidence from human work on drastic surgery-induced weight loss (38) versus short-term weight loss (42), the duration of the maternal treatment phase may have been too short. Although we did treat the rats for 8 weeks, a longer period of time might have allowed for sufficient weight loss to result in improved fecundity outcomes. Furthermore, we used targeted qCR for our gut microbiota analysis to examine bacterial groups relevant to obesity but it is plausible that global profiling of the microbiota using 16S rRNA sequencing would reveal alterations in the abundance of other bacterial groups. Finally, Togashi et al. (63) recently demonstrated that glucometers, although widely used in animal model research, typically yield higher glucose values than glucose measured with laboratory tests in plasma. Therefore, our blood glucose results likely reflect higher values than if they were measured with a mutarotase GOD method.

Based on the results of this study it can be concluded that small amounts of weight loss before pregnancy may have some lasting beneficial effects on offspring that appear to be sex specific including adiposity in males and glucose tolerance in females. The magnitude of weight loss achieved prepregnancy, however, was not associated with any improvement in fecundity. While dietary intervention with OFS *during* pregnancy and lactation has previously been shown to alter maternal gut microbiota substantially (21, 49), intervening before pregnancy with OFS or sitagliptin did not have any lasting effects that could be detected at birth beyond increased *C*. cluster I and XI in Obese-S dams which mimicked Obese-HFS dams. Offspring of untreated Obese-HFS dams, however, displayed dysbiosis in terms of lower *Bacteroides*/*Prevotella* and *Lactobacillus* which seemed to be partially corrected by weight loss intervention preconception. More research, at the basic and clinical level, is warranted to determine specific weight loss guidelines for women dealing with obesity-related infertility.

## Ethics Statement

Ethical approval for the study was granted by the University of Calgary Life and Environmental Science Animal Care Committee and conformed to the Canadian Council on Animal Care guidelines.

## Author Contributions

CD and AE were responsible for data acquisition. CD was responsible for data analysis and interpretation and drafting the manuscript. RR was responsible for the study conception and design, data analysis, and interpretation. All the authors critically reviewed the work and approved the final version.

## Conflict of Interest Statement

CD and AE declare no conflict of interest. RR previously held a research contract from Beneo, manufacturer of P95 oligofructose, for work unrelated to this study.
